# Scent dogs identify SARS-CoV-2-infections in respiratory samples from experimentally infected ferrets and hamsters—a pilot study

**DOI:** 10.3389/fmed.2024.1476300

**Published:** 2024-12-09

**Authors:** Claudia Schulz, Friederike Twele, Sebastian Meller, Nele A. ten Hagen, Veronika Pilchová, Katrin Wirz, Sabrina Clever, Christian Meyer zu Natrup, Asisa Volz, Maren von Köckritz-Blickwede, Holger A. Volk

**Affiliations:** ^1^Research Center for Emerging Infections and Zoonoses (RIZ), University of Veterinary Medicine Hannover, Hannover, Germany; ^2^Department of Small Animal Medicine and Surgery, University of Veterinary Medicine Hannover, Hannover, Germany; ^3^Institute of Biochemistry, University of Veterinary Medicine Hannover, Hannover, Germany; ^4^Institute of Virology, University of Veterinary Medicine Hannover, Hannover, Germany

**Keywords:** scent dog, COVID-19, SARS-CoV-2, diagnosis, animal experiment, ferret, hamster

## Abstract

Rapid and sensitive diagnostic measures are a pre-requisite for the control of SARS-CoV-2 outbreaks. Dogs detect SARS-CoV-2-infected human individuals with high speed due to their extraordinary olfactory acuity. In the post-pandemic phase of SARS-CoV-2 it is difficult to obtain samples from infected humans for scent dog training. Established animal models for COVID-19 include hamsters and ferrets, which could overcome this shortcoming and have the advantage that samples are generated under controlled conditions. Respiratory samples from humans, hamsters and ferrets infected with SARS-CoV-2 and from ferrets infected with an H7-Influenza A virus were inactivated with *β*-propiolactone and presented via a device called “Detection Dog Training System” (DDTS). DDTS allows a fast, blinded, randomized, and automated sample presentation without trainer interference. Scent dogs generally showed a similar diagnostic sensitivity (DSe) and specificity (Dsp) for four tested scenarios (S1-4) and as reported previously for respiratory samples from humans. (S1) Human with COVID-19: DSe 88.1 [74.0–100.0% CI_95%_] and DSp 89.6 [80.6–98.5% CI_95%_]. (S2) Hamster with COVID-19: DSe 82.4 [74.1–90.7% CI_95%_] and DSp 96.7 [93.7–99.7% CI_95%_]. (S3) Ferret with COVID-19: DSe 86.2 [69.8–100.0% CI_95%_] and DSp 95.1 [89.5–100.0% CI_95%_]. (S4) Ferrets infected with an H7 Influenza A-virus (IAV) as a distractor: DSe 96.9 [57.2–100.0% CI_95%_] and DSp 89.86 [40.3–100.0% CI_95%_]. We provide evidence that scent dogs detect samples from SARS-CoV-2-infected hamsters and ferrets with a similar accuracy as reported for humans. The study highlights that volatile organic compound odor patterns are similar in humans, hamsters, and ferrets after SARS-CoV-2 infection, but distinct after IAV-infection.

## 1 Introduction

Severe acute respiratory syndrome coronavirus 2 (SARS-CoV-2) emerged in 2019 and caused severe illness and substantial mortality in humans. Early detection of SARS-CoV-2-infected individuals is the most crucial control measure for interrupting the chain of infection. Several studies have already demonstrated the extraordinary olfactory acuity and speed of dogs in detecting SARS-CoV-2-infected individuals through disease-specific odor patterns derived from volatile organic compounds (VOCs) in different body fluids from humans. The diagnostic accuracies of scent dogs are comparable with SARS-CoV-2-specific real-time quantitative reverse transcription-PCR (RT-qPCR) (1–4).

A crucial step during training is to achieve the right balance between olfactory generalization and discrimination of odor patterns. Therefore, a diverse range of samples from varying symptomatic and asymptomatic COVID-19 patients at different stages of the disease is essential. Furthermore, in the post-pandemic, i.e., endemic, phase of SARS-CoV-2, it is difficult to obtain samples from infected humans. Established animal models of pathophysiological states for COVID-19 include hamsters and ferrets (5), which could overcome this shortcoming. Furthermore, if canines are able to scent SARS-CoV-2 infections in hamsters and ferrets, this would strengthen the translational character of these animal models.

## 2 Methods

### 2.1 Acquisition, analysis, and inactivation of samples from humans and animals for SARS-CoV-2 and Influenza A virus detection

Sputum, sweat and urine samples from humans previously diagnosed with COVID-19 based on SARS-CoV-2-RNA detection or from healthy volunteers were collected during the COVID-19 pandemic from 2020 to 2021 (Table 1). Hamster and ferret samples were re-used from animal experiments that were conducted for other research purposes. The animal husbandry, health monitoring, study design, experimental procedures (including for example anesthesia regimens, experimental infection, sample collection, clinical monitoring, human endpoint criteria and euthanasia) were approved by an independent ethical committee (see also section Ethical statement) and conducted as described previously (5–8). Hamsters were inoculated intranasally with phosphate buffered saline (PBS) or infected with 10^4 TCID_50_/ml SARS-CoV-2 (7) and sacrificed at 6 days post infection (dpi). Ferrets were infected intratracheally with 1 mL of 10^6 TCID_50_/ml SARS-CoV-2 (5) or with 1.5 mL of 10^5 or 10^6 plaque forming units (PFU) of an H7-Influenza A virus (IAV) or with PBS (8). Oropharyngeal swabs and nasal swabs were collected before and at 4, 7, 14 or 21 dpi after infection, while bronchioalveolar lavage fluid (BALF) samples were collected immediately after euthanasia. Specific-pathogen free ferrets served as negative control animals (5–8). An overview of the samples is provided in Table 1.

**Table 1:** Overview of samples for scent dog detection by scenario.

Scenario	Study ID	SARS-CoV-2 status	IAV status	Material	Number of samples	Dpi (SARS-CoV-2)	Dpi (IAV)
1	Human SARS-CoV-2	pos	–	Sputum	2		
		pos	–	Sweat	2		
		pos	–	Urin	3		
		neg	–	Sputum	4		
		neg	–	Sweat	1		
		neg	–	Urin	2		
2	Hamster SARS-CoV-2	pos	–	BALF supernatant	4	6	
		pos	–	OP swab	3	6	
		neg	–	BALF supernatant	4	None	
		neg	–	OP swab	3	None	
3	Ferret SARS-CoV-2	pos	neg	BALF supernatant	3	4, 7, 21	
		pos	neg	OP swab	4	4, 7, 14, 21	
		neg	neg	BALF supernatant	2	None	
		neg	neg	OP swab	3	None	
		neg	neg	Nasal swab	2	None	
4	Ferret SARS-CoV-2versus IAV	pos	neg	BALF supernatant	3	4, 7, 21	
		pos	neg	OP swab	4	4, 7, 14, 21	
		neg	neg	OP swab	3	None	
		neg	pos	Nasal swab	4	None	4, 7, 14, 21

OP swab, oropharyngeal swab; BALF, bronchioalveolar lavage fluid; Dpi, days post infection; IAV, H7 Influenza A virus; pos, positive; neg, negative; −, not analysed.

SARS-CoV-2-RNA in human and animal samples (1, 5) and H7-IAV-RNA (8, 9) in ferret samples were analyzed by real-time quantitative reverse transcription-PCR (RT-qPCR) with an internal control system (pcDNA3-EGFP, Plasmid #13031, Addgene) as described previously (1, 5, 8, 9). Briefly, 100 μL was extracted at a KingFisher 96 (Thermo Fisher Scientific) using the NucleoMagVet kit (Macherey-Nagel). A total of 2.5 μL eluate was used for amplification of SARS-CoV-2-RNA or H7-IAV-RNA using the AgPath-ID™ One-Step RT-PCR Reagent kit (Thermo Fisher Scientific, 4,387,424) at a CFX96 real-time PCR detection system (Bio-Rad) or a AriaMx Real-Time PCR System (Agilent), respectively. The RT-qPCR temperature protocols included reverse transcription for 10 min at 45°C, activation Taq for 10 min at 95°C, and 42 cycles of denaturation for 15 s at 95°C, annealing for 20 s at 57°C (SARS-CoV-2) or 56°C (H7-IAV) and elongation for 30 s at 72°C. Positive and negative controls were included in each RT-qPCR run.

The forward and reverse primers and the probes used for the H7-IAV RT-qPCR and SARS-CoV-2 assays including the EGFP assay as internal control system were as followed (sequences given *5′-3′*): AYA GAA TAC AGA TWG ACC CAG T (IAV-HA7-1593-F), TAG TGC ACY GCA TGT TTC CA (IAV-HA7-1740-R), *FAM-*TGG TTT AGC TTC GGG GCA TCA TG-*BHQ1* (AIV-HA7-1649-FAM) (8, 9); GGT AAC TGG TAT GAT TTC G (SARS2-IP4-14059F), CTG GTC AAG GTT AAT ATA GG (SARS2-IP4-14146R), *FAM-*TCA TAC AAA CCA CGC CAG G*-BHQ1* (SARS2-IP4-14084FAM); GAC CAC TAC CAG CAG AAC AC (EGFP-1-F), GAA CTC CAG CAG GAC CAT G (EGFP2-F), *HEX-*AGC ACC CAG TCC GCC CTG AGC A*-BHQ1* (EGFP-HEX) (1, 5).

### 2.2 Beta-propiolactone (BPL) inactivation of respiratory samples

**Figure 1:**
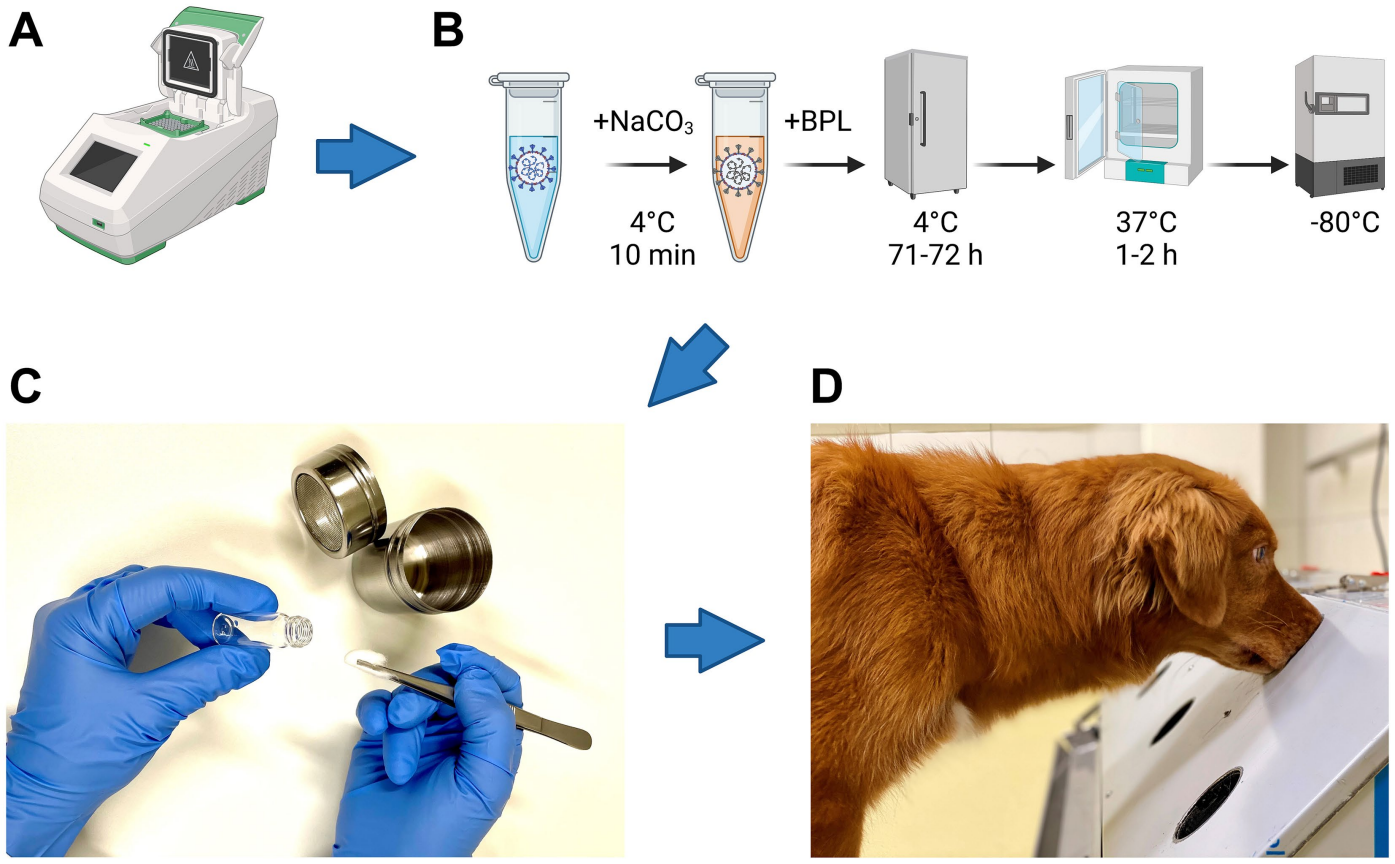
Flow chart of sample analyses. **(A)** Real-time reverse transcription-PCR analysis of samples to detect SARS-CoV-2-RNA and Influenza A virus (IAV)-RNA. **(B)** Five steps required for inactivation of SARS-CoV-2 and IAV with beta-propiolactone (BPL): samples were (i) buffered with 10% NaHCO_3_ and incubated for 10 min at 4°C, (ii) inactivated with BPL for 71 to 72 h at 4°C, (iii) BPL was hydrolyzed for 1 to 2 h at 37°C and (iv) stored at−80°C until presentation to the dogs for training and study purposes. **(C)** Sample preparation for a device called “Detection Dog Training System” (DDTS). **(D)** Sample presentation to dogs via DDTS. Icons in pannels **(A,B)** were created with Biorender.com (Agreement number FD26B6H2CJ).

All samples were chemically inactivated with beta-propiolactone (BPL) according to Pilchová et al. (10) as shown in Figure 1B.

### 2.3 Test scenarios and scent dog detection of inactivated SARS-CoV-2 and influenza samples

**Figure 2:**
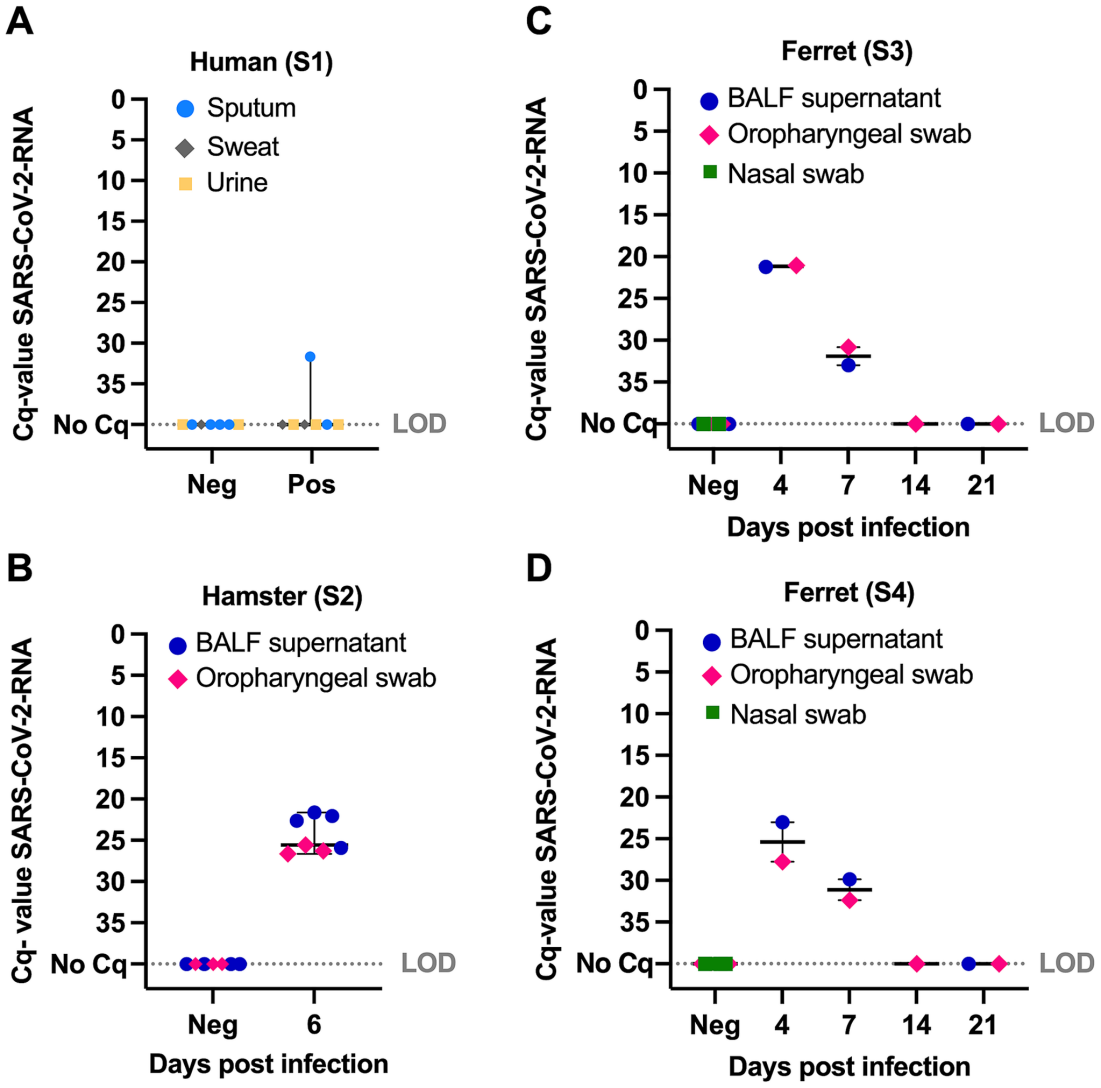
Quantitative cycle values (Cq) of SARS-CoV-2-RNA detected by real-time reverse transcription-PCR in samples from humans, hamsters and ferrets by scenario (S) S1 to S4, species and sample matrix. **(A)** SARS-CoV-2-RNA in sputum, sweat and urine from human COVID-19 patients that were tested positive for SARS-CoV-2 at an earlier time point by a clinic or diagnostic laboratory (S1). **(B)** SARS-CoV-2-RNA in samples collected from hamsters before infection and at 6 days post infection (dpi) (S2). **(C)** SARS-CoV-2-RNA in samples collected from ferrets before infection and at 4 to 21 dpi (S3). **(D)** SARS-CoV-2-RNA in samples from ferrets as described for S3, except that in S4 dogs were additionally presented samples from ferrets infected with H7 influenza A virus as distractor. BALF, bronchioalveolar lavage fluid; LOD, limit of detection. Whiskers show median with 95% confidence intervals.

Three dogs that were previously trained solely with samples from human COVID-19 patients (scenario 1, Table 1) were confronted for the first time with respiratory samples collected from hamsters (scenario 2) and ferrets (scenario 3 and 4) at different days before and after SARS-CoV-2 infection. In scenario 4, ferrets infected with an IAV (8) were included as distractor samples (Table 1). In scenario 4, only two of three dogs were available. Samples collected after infection were considered as “positive” irrespective of the PCR-result (Tables 1, 2; Figure 2).

**Table 2:** Diagnostic sensitivity (DSe) and specificity (DSp) for the detection of SARS-CoV-2 infection in human and animal samples by scent dogs.

Scenario	Study ID	Dog#	Detection	No. of samples			Diagnostic sensitivity		Diagnostic specificity	
				SARS-CoV-2 positive	SARS-CoV-2 negative	Total	%	95% CI	%	95% CI
1	Human SARS-CoV-2	Dog 1	Yes	15	12	100	88.24	63.56–98.54	85.54	76.11–92.30
			No	2	71					
		Dog 2	Yes	14	8	102	82.35	56.57–96.20	90.59	82.29–95.85
			No	3	77					
		Dog 3	Yes	15	5	83	93.75	96.77–99.84	92.54	83.44–97.53
			No	1	62					
		Total		50	235	285	88.11	73.95–100.00	89.56	80.58–98.53
2	Hamster SARS-CoV-2	Dog 1	Yes	14	2	74	87.50	61.65–98.45	96.55	88.09–99.58
			No	2	56					
		Dog 2	Yes	13	4	80	72.22	46.52–90.31	93.55	84.30–98.21
			No	5	58					
		Dog 3	Yes	14	0	73	87.50	61.65–98.45	100.00	93.73–100.00
			No	2	57					
		Total		50	177	227	82.41	74.13–90.69	96.70	93.67–99.73
3	Ferret SARS-CoV-2	Dog 1	Yes	14	4	72	82.35	56.57–96.20	92.73	82.41–97.98
			No	3	51					
		Dog 2	Yes	14	3	83	82.35	56.57–96.20	95.45	87.29–99.05
			No	3	63					
		Dog 3	Yes	15	2	88	93.75	69.77–99.84	97.22	90.32–99.66
			No	1	70					
		Total		50	193	243	86.15	69.80–100.00	95.13	89.51–100.00
4	Ferret SARS-CoV-2 versus IAV/negative	Dog 1	Yes	15	12	86	100.00	78.20–100.00	83.10	72.34–90.95
			No	0	59					
		Dog 2	Yes	15	2	75	93.75	69.77–99.84	96.61	88.29–99.59
			No	1	57					
		Total		31	130	161	96.77	83.81–99.83	89.23	82.73–93.48

DSe, DSp and 95% confidence intervals (95% CI) were calculated using GraphPad prism (version 9).

All samples were presented via a device called “Detection Dog Training System” (DDTS) (1) (Figure 1). DDTS allows a fast, blinded, randomized and automated sample presentation without trainer interference (1).

Statistical analysis was calculated with GraphPad prism (version 9), and the accuracy with Medcalc statistical software.^1^

### 2.4 Ethical statement

Human participation was approved by ethics committee of Hannover Medical School with consent number 9042_BO_K_2020 and 9940_BO_S_2021. Animal experiments were approved by an independent ethical committee (Niedersächsisches Landesamt für Verbraucherschutz und Lebensmittelsicherheit, LAVES) and registered with the permission numbers 20-3482 (hamster), 2-3402 (SARS-CoV-2, ferrets), 20-3499 (IAV, ferrets).

## 3 Results

SARS-CoV-2-RNA in human samples (scenario 1) was detected in one (quantitative cycle values (Cq) 31.7) of seven tested samples from COVID-19 patients that were tested positive for SARS-CoV-2 at an earlier time point by a clinic or diagnostic laboratory. S2: All samples collected from hamsters (scenario 2) at 6 dpi or ferrets (scenarios 3 and 4) at 4 to 7 dpi were positive for SARS-CoV-2-RNA (Cq 21.1 to 33.0), while no SARS-CoV-2-RNA was detected in ferret samples collected at 14 dpi, 21 dpi or before infection (Figure 2). IAV was detected in one nasal swab from one ferret collected at 4 dpi (Cq 28.6), while the nasal swabs collected at 7, 14 and 21 dpi were RT-qPCR-negative.

Scent dogs generally showed a similar diagnostic sensitivity (DSe) and specificity (Dsp) for four tested scenarios (S1-4) (Table 2). (S1) Human with COVID-19: DSe 88.1 [74.0–100.0% CI_95%_] and DSp 89.6 [80.6–98.5% CI_95%_]. (S2) Hamster with COVID-19: DSe 82.4 [74.1–90.7% CI_95%_] and DSp 96.7 [93.7–99.7% CI_95%_]. (S3) Ferret with COVID-19: DSe 86.2 [69.8–100.0% CI_95%_] and DSp 95.1 [89.5–100.0% CI_95%_]. (S4) Ferrets infected with an H7 Influenza A-virus (IAV) as a distractor: DSe 96.9 [57.2–100.0% CI_95%_] and DSp 89.86 [40.3–100.0% CI_95%_].

Accordingly, scent dogs generally showed a similar accuracy (89–94%) (see details in Supplementary Table S1), DSe (82–97%) and DSp (90–97%) for all four scenarios, but the DSe in scenario 4 was noticeable higher (97%) compared to scenarios 1–3 (82–89%) (see details in Table 2; Supplementary Figure S1). In scenario 4, the DSe and DSp generally showed wider 95% confidence intervals compared to scenarios 1–3 (Table 2; Supplementary Figure S1). The negative predictive values (NPV) were similar for all four scenarios (95–99%), while the PPV varied between scenarios 1 and 4 (65 and 68%) in comparison to scenarios 2 and 3 (88 and 83%) (see details in Supplementary Table S2). Fisher exact tests were significant (*p* < 0 0.0001) for all trials, by dog and scenario (Supplementary Table S1).

## 4 Discussion

Thanks to the excellent olfactory acuity of canines, scent dogs detect various odors of explosives and drugs, odors produced during disease manifestation including metabolic disorders such as different types of cancer, hyperglycemia, and VOCs released after infection with different pathogens (11, 12). During the COVID-19 pandemic, scent dogs demonstrated a high sensitivity and specificity to rapidly diagnose COVID-19 in humans (1, 13). We present here the first study to demonstrate that scent dogs detect VOC release after SARS-CoV-2 infection in samples other than humans, from animal species, such as ferrets and hamsters, with previous training solely on human samples. The accuracy, DSe and DSp of scent dogs to positively decide for different respiratory sample matrices from SARS-CoV-2 infected hamsters and ferrets after training with human samples was similar to the accuracy, DSe and DSp in human samples in our study (Table 2; Supplementary Table S1) and as reported for respiratory samples from humans before (1–3, 13). The results highlight that COVID-19 detection dogs differentiate between samples from infected and non-infected ferrets and hamsters without specific prior training with animal samples and suggests that the VOCs in human samples and laboratory animals are very similar independent from the host species. These findings strengthen the use of ferrets and hamsters as animal models for human SARS-CoV-2 infections and as a source for future canine training materials. Furthermore, comparison of VOCs in headspace air samples from humans (14, 15) and animals infected with SARS-CoV-2 and other respiratory virus using solid-phase microextraction (SPME) and gas chromatography/mass spectrometry (GC/MS) methods might illuminate species-unspecific VOCs characteristic for COVID-19. Interestingly, VOCs from animals and humans generally differ as reported for muscle tissue (16) or decomposed animals (17) and humans using SPME and GC/MS or “Human Remains Detection” dogs (16, 17).

The successful training to detect VOC-specific odor patterns may be influenced by environmental factors (11). Training with samples from experimentally infected laboratory animals could overcome challenges associated with obtaining human samples and enhance the efficiency of training detection dogs, not only for COVID-19 but also for future pandemics. Additionally, this offers novel opportunities to train dogs with a large number and variety of high-quality samples with target odors that were generated under controlled conditions. For ethical reasons and in favor of the 3R-principle to reduce, refine and replace animal experiments, no animal experiments are required to generate samples for the purpose of scent dog training (18). Collaborative networks and biobanks facilitate rapid access not only to human patient samples but also to animal samples from experiments conducted under controlled conditions, which provide comprehensive data including pathogens and disease progression (19, 20).

Experimentally infected animals generally show an infection rate of IAV and SARS-CoV-2 of about 100%. Viral shedding ceases in respiratory samples after the peak of IAV and SARS-CoV-2 infection during the first to second week after infection due to virus elimination by the cellular and humoral immune responses elicited by the innate and adaptive immune systems (6–8, 21–23). Therefore, IAV-RNA and SARS-CoV-2-RNA was detected in respiratory samples until 4 or 7 dpi (Figure 2), respectively.

The results of our pilot study (scenario 2–4), indicate that dogs show a lower percentage of false negative decisions in samples collected during the first week (hamster: 3.4%; ferret: 5.0%) compared to 14 or 21 dpi (ferret: 20–21%) after SARS-CoV-2 infection. In contrast, the percentage of false positive decisions was generally higher after infection and varied between 12 and 33% (Supplementary Table S3). On the other hand, the detection of SARS-CoV-2-RNA-negative samples as positive after the peak of infection (14 and 21 dpi) indicate that “post-COVID-19” infection can also be detected by scent dogs in ferrets as previously reported for post-COVID-19 patients (3, 24).

In scenario 4, the DSe and DSp showed wider 95% confidence intervals compared to scenarios 1–3 (Table 2; Supplementary Figure S1). This can be explained by the lower number of sample presentations compared to scenarios 1–3, since only two of three dogs could be used for the trial, due to other deployments of the dogs and their handlers at the time of our study. Both dogs were able to distinguish between IAV and SARS-CoV-2 positive samples, although the proportion of false positive decisions varied between the two dogs (13% versus 2%), indicating that the training results depend on the individual dog (Supplementary Table S4). Furthermore, training with distractory samples including other respiratory pathogens to distinguish from SARS-CoV-2 positive samples is essential to optimize the DSp as suggested previously (13).

The advantage to use scent dogs in disease diagnosis generally include fast detection, high sensitivity, short training period and application for large-scale screening events at low costs. Hence, scent dog detection may be applied to individual diagnosis, but also at large-scale, for example in a pandemic situation. The disadvantages to use scent dogs is that the training and performance characteristics of scent dogs rely on various factors. The training success relies on the handler and training efficiency, which often lacks a certified standardized and validated process. Furthermore, intrinsic (e.g., anatomy, health, behavior, age) and extrinsic factors (experience, operational environment) may influence the dogs performance. The selection of the right target samples and sample diversity is essential for the training of dogs to discriminate between the target and background scent required for olfactory generalization. Last but not least, ethical considerations are paramount to ensure that the used training methods and materials are in accordance with animal welfare, the current state of research and do not harm the dogs health integrity (11, 12, 25, 26).

In the present study, we applied previously approved and standardized training methods that resulted in a high DSe and DSp to detect VOCs specific for SARS-CoV-2 infection in different matrices of samples from human COVID-19 patients. The study results confirmed that the training methods can also be successfully applied on the detection of VOCs specific for SARS-CoV-2 infection in animal samples from ferrets and hamsters. Furthermore, the study protocols were approved by an independent ethical committee in accordance with animal welfare and the samples were inactivated with BPL to preclude infection of dogs with SARS-CoV-2.

## 5 Conclusion

The study highlights that the VOC odor pattern released from established animal models for SARS-CoV-2 is similar to human VOCs (1–3, 13), independent from the sample matrix and distinct from VOCs released after IAV-infection. In favor of the principle of sustainability, our protocol can be adapted to future pandemics with other pathogens.

## Data Availability

The original contributions presented in the study are included in the article/Supplementary material, further inquiries can be directed to the corresponding author.

## Footnotes

^1^ https://www.medcalc.org/calc/diagnostic_test.php
