# Supplementary material for: Scent dogs identify SARS-CoV-2-infections in respiratory samples from experimentally infected ferrets and hamsters—a pilot study
Source: Front Med (Lausanne). 2024 Dec 9;11:1476300. doi: 10.3389/fmed.2024.1476300 (PMC11666280; doi:10.3389/fmed.2024.1476300)
Supplement: Supplementary file 1 [file Data_Sheet_1.PDF]

Supplementary Material

Scent dogs identify SARS-CoV-2-infections in respiratory samples from experimentally infected ferrets and hamsters – a pilot study

Claudia Schulz[1#†], Friederike Twele[2†], Sebastian Meller[2], Nele A. ten Hagen[2], Veronika Pilchová[1,3$], Katrin Wirz[1,3], Sabrina Clever[1,4], Christian Meyer zu Natrup[1,4], Asisa Volz[1,4], Maren von Köckritz-Blickwede[1,3], and Holger A. Volk[2*]

1 Supplementary Figure

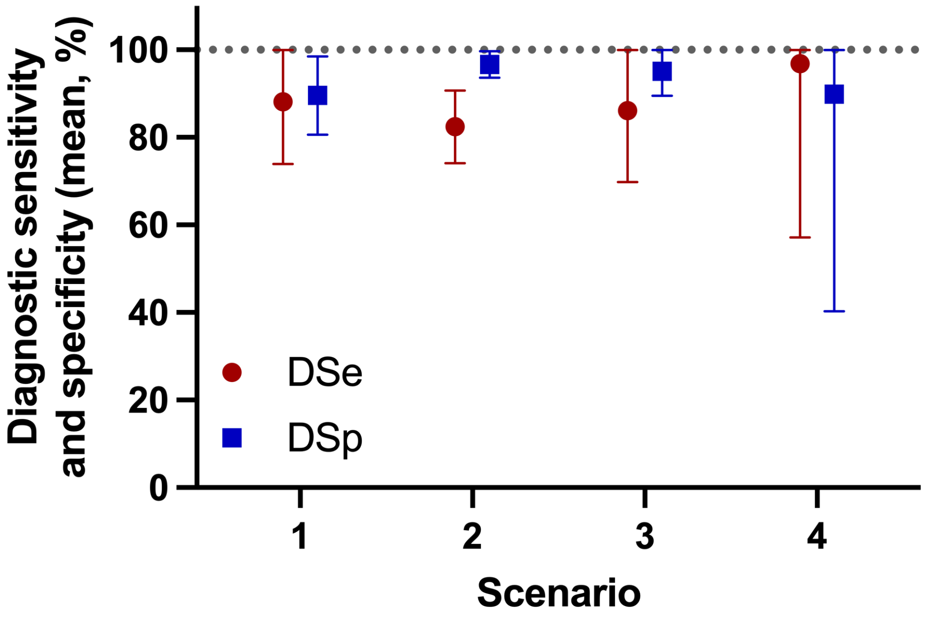

Supplementary Figure 1. The mean diagnostic sensitivity (DSe) and specificity (DSp) of scent dogs comparing four different scenarios (S1 to S4) after training with human samples (S1: DSe 88.11%, DSp 89.56) from COVID-19 patients and healthy controls to detect samples from SARS-CoV-2 infected hamsters (S2: DSe 82.41%, DSp 96.70%) and ferrets without (S3: DSe 86.15%, DSp 95.13%) or with samples from Influenza A infected ferrets as distractor (S4: DSe 96.88%, DSp 89.86%). Whiskers represent 95% confidence intervals.

2 Supplementary Tables

Supplemental Table S1. Accuracy for the detection of SARS-CoV-2 infection in human and animal samples by scent dogs. Accuracy, 95% confidence intervals (95% CI) and Fisher exact test p values were calculated with Medcalc statistical software (https://www.medcalc.org/calc/diagnostic_test.php) and GraphPad prism (version 9), respectively.

Scenario	Study ID	Dog#	Detection	No. of samples			Accuracy		Fisher exact test *p*-value
				SARS-CoV-2 positive	SARS-CoV-2 negative	Total	%	95% CI	
**1**	**Human**	Dog 1	Yes	15	12	100	86.00	77.63-92.13	****
	**SARS-CoV-2**		No	2	71				
		Dog 2	Yes	14	8	102	98.22	81.52-94.49	****
			No	3	77				
		Dog 3	Yes	15	5	83	92.77	84.93-97.30	****
			No	1	62				
		**Total**		**50**	**235**	**285**	**89.33**	**80.92-97.74**	****
**2**	**Hamster**	Dog 1	Yes	14	2	74	94.59	86.73-98.51	****
	**SARS-CoV-2**		No	2	56				
		Dog 2	Yes	13	4	80	88.75	79.72-94.72	****
			No	5	58				
		Dog 3	Yes	14	0	73	97.26	90.45-99.67	****
			No	2	57				
		**Total**		**50**	**177**	**227**	**93.53**	**89.45-97.62**	****

**** $p < 0.0001$

Supplemental Table S1 continued.

Scenario	Study ID	Dog#	Detection	No. of samples			Accuracy		Fisher exact test *p*-value
				**SARS-CoV-2 positive**	**SARS-CoV-2 negative**	**95% CI**	**%**	**95% CI**	
**3**	**Ferret**	Dog 1	Yes	14	4	72	90.28	80.99-96.00	****
	**SARS-CoV-2**		No	3	51				
		Dog 2	Yes	14	3	83	92.77	84.93-97.30	****
			No	3	63				
		Dog 3	Yes	15	2	88	96.59	90.36-99.29	****
			No	1	70				
		**Total**		**50**	**193**	**243**	**93.21**	**85.32-100.00**	****
**4**	**Ferret**	Dog 1	Yes	15	12	86	86.05	76.89-92.58	****
	**SARS-CoV-2**		No	0	59				
	**versus IAV/ negative**	Dog 2	Yes	15	2	75	96.00	88.75-99.17	****
			No	1	57				
		**Total**		**31**	**130**	**161**	**90.68**	**85.10-94.69**	****

**** $p < 0.0001$

Supplemental Table S2. Positive (PPV) and negative predictive values (NPV) for the detection of SARS-CoV-2 infection in human and animal samples by scent dogs. PPV, NPV and 95% confidence intervals (95% CI) were calculated using GraphPad prism (version 9).

Scenario	Study ID	Dog#	Detection	No. of samples			PPV		NPV	
				SARS-CoV-2 positive	SARS-CoV-2 negative	Total	%	95% CI	%	95% CI
**1**	**Human**	Dog 1	Yes	15	12	100	55.56	35.55-74.52	97.26	90.45-99.67
	**SARS-CoV-2**		No	2	71					
		Dog 2	Yes	14	8	102	63.64	40.66-82.80	96.25	89.43-99.22
			No	3	77					
		Dog 3	Yes	15	5	83	75.00	50.90-91.34	98.41	91.47-99.96
			No	1	62					
		**Total**		**50**	**235**	**285**	**64.73**	**40.47-88.99**	**97.31**	**94.62-99.99**
**2**	**Hamster**	Dog 1	Yes	14	2	74	87.50	61.65-98.45	96.55	88.09-99.58
	**SARS-CoV-2**		No	2	56					
		Dog 2	Yes	13	4	80	76.47	50.10-93.19	92.06	82.44-97.37
			No	5	58					
		Dog 3	Yes	14	0	73	100.00	76.84-100.00	96.61	88.29-99.59
			No	2	57					
		**Total**		**50**	**177**	**227**	**87.99**	**76.94-99.04**	**95.08**	**92.62-97.52**

Supplemental Table S2 continued.

Scenario	Study ID	Dog#	Detection	No. of samples			PPV		NPV	
				SARS-CoV-2 positive	SARS-CoV-2 negative	Total	%	95% CI	%	95% CI
**3**	**Ferret**	Dog 1	Yes	14	4	72	77.78	52.36-93.59	94.44	84.61-98.84
	**SARS-CoV-2**		No	3	51					
		Dog 2	Yes	14	3	83	82.35	56.57-96.20	95.45	87.29-99.05
			No	3	63					
		Dog 3	Yes	15	2	88	88.24	63.56-98.54	98.59	92.40-99.96
			No	1	70					
		**Total**		**50**	**193**	**243**	**82.79**	**69.76-95.82**	**96.16**	**90.78-100.00**
**4**	**Ferret**	Dog 1	Yes	15	12	86	55.56	37.31-72.41	100.00	93.89-100.00
	**SARS-CoV-2**		No	0	59					
	**versus IAV/ negative**	Dog 2	Yes	15	2	75	88.24	65.66-97.91	98.28	90.86-99.91
			No	1	57					
		**Total**		**31**	**130**	**161**	**71.90**	**53.44-80.00**	**99.15**	**95.32-99.96**

Supplemental Table S3. Number of false positive (A) and false negative (B) decisions by dogs, depending on sampling time point (scenarios 3 and 4). Dpi: days after SARS-CoV-2 infection.

**A)**		**4 dpi**			**7 dpi**			**14 dpi**			**21 dpi**			**Blank trial**		
	**Dog ID**	**Total**	**False positive**	**%**	**Total**	**False positive**	**%**	**Total**	**False positive**	**%**	**Total**	**False positive**	**%**	**Total**	**False positive**	**%**
Scenario 3	Dog 1	5	0	0	4	2	50	2	0	0	4	2	50	55	0	0
	Dog 2	6	0	0	2	0	0	4	1	25	3	0	0	66	2	3
	Dog 3	5	0	0	1	0	0	4	1	25	5	1	20	72	0	0
	**All dogs**	16	0	**0**	7	2	**29**	10	2	**20**	12	3	**25**	193	2	**1**
Scenario 4	Dog 1	5	2	40	4	3	75	1	0	0	5	2	40	71	5	7
	Dog 3	4	1	25	4	0	0	4	0	0	3	0	0	59	1	2
	All dogs	9	3	**33**	8	3	**38**	5	0	**0**	8	2	**25**	130	6	**5**
**Scenario 3+4**	**All dogs**	25	3	**12**	15	5	**33**	15	2	**13**	20	5	**25**	323	8	2

**B)**		**4 dpi**			**7 dpi**			**14 dpi**			**21 dpi**		
	**Dog ID**	**Total**	**False negative**	**%**	**Total**	**False negative**	**%**	**Total**	**False negative**	**%**	**Total**	**False negative**	**%**
Scenario 3	Dog 1	5	0	0	4	0	0	2	0	0	4	3	75
	Dog 2	6	2	33	2	0	0	4	1	25	3	0	0
	Dog 3	5	0	0	1	0	0	4	1	25	5	0	0
	**All dogs**	16	2	**13**	7	0	**0**	10	2	**20**	12	3	**25**
Scenario 4	Dog 1	5	0	0	4	0	0	1	0	0	5	0	0
	Dog 3	4	0	0	4	0	0	4	1	25	3	0	0
	All dogs	9	0	**0**	8	0	**0**	5	1	**20**	8	0	**0**
**Scenario 3+4**	All dogs	25	2	**8**	15	0	**0**	15	3	**20**	20	3	**15**

Supplemental Table S4. Number of false positive decisions for SARS-CoV-2 infection in ferrets by dogs that were distracted with samples from negative or H7-Influenza A virus (IAV)-infected ferrets (scenario 4).

	**IAV**			**Negative Samples**		
	**Total**	**False positive**	**%**	**Total**	**False positive**	**%**
**Dog 1**	54	7	13	43	5	12
**Dog 3**	57	1	2	40	1	3
**Dog 1+3**	111	8	7	83	6	7
